# The King's Fund and the Government Grant

**Published:** 1921-07-23

**Authors:** 


### The Hospital

The Workers' Newspaper of Administrative Medicine and Institutional Life. Administration. National Insurance and Health.

No. 1833, Vol. LXX. SATURDAY, JULY 23, 1921. PRICE TWOPENCE

### THE KING'S FUND AND THE GOVERNMENT GRANT.

The two important events of last week were the special meeting at St. James's Palace of the General Council of King Edward's Hospital Fund for London on the 13th instant and the publication of the terms of appointment of the Voluntary Hospitals Commission, which we reproduce on the next page. It is necessary to relate these two events, because it is clear that the recommendations passed at the meeting of the King's Fund were necessarily influenced by the Government's instructions to the Commission appointed on the recommendation of Lord Cave's Committee. What, then, is the official policy? The Voluntary Hospitals Commission will act on the following assumptions: The Government favours the continuance of the voluntary system, reluctantly grants a special contribution to meet the difficulties of the moment only, but declines to make good the present general deficit; in other words, the grant of half a million which the Commission will distribute is only to give the voluntary system a moment's breathing-time. The system is otherwise left to save itself. A great deal, therefore, must be done in a limited time, hence the recommendations of the King's Fund, which, as we shall see, are made matters of urgency.

It is in the light of the official programme that the recommendations of King Edward's Hospital Fund must be understood. In moving them Lord Stuart of Wortley declared that the £500,000 suggested by the Government is clearly not enough. The recommenders thank Lord Cave's Committee for its suggestions, and suggest that the next two years must be regarded as an emergency, during which "a Government grant on a sufficiently substantial scale will be necessary." They point out that for 1921 the recommended grant of a million will probably be required, since "the deficits at the London hospitals alone are likely to amount to about half this sum." They emphasise the fact that "many of the hospitals are at present carried on at a large weekly deficit, and that the position of hospitals throughout the country is thereby imperilled," and finally urge the danger of even a few weeks' delay in carrying out the recommendations of Lord Cave's Committee if immediate disaster, and consequent expense to the Exchequer, are to be avoided. Now, granted that these recommendations are mainly recapitulations of familiar facts, their meaning is seen in the decision to send copies of them to the Prime Minister, the Chancellor of the Exchequer, the Minister of Health, and, of course, to the members of Lord Cave's Committee. The Government is warned again of the position, for, on the one hand, the King's Fund turns to the Government and explains that the promised grant is inadequate to the gravity of the situation; on the other, by a subsequent resolution, it informs the London hospitals that the Fund will consider, when making its own distribution, the activity of each hospital in carrying out the Cave Commission's suggestions. The hospitals who wish to qualify for sympathetic hearing of their needs must therefore show the greatest efforts to reorganise their incomes in accordance with one or other of the three alternative schemes. An account of one of these, the "Sussex Scheme," has already appeared in our columns, and to-day we publish a description of the plan and working of another, the "Oxford Scheme."

It is hard to see what more the King's Fun[illegible] can do with the limited time and the limite[illegible] resources at its disposal. Whether the Government will be moved by these recommendations to admit the inadequacy of its grant remains to be seen. No hopes can be built upon it. The King's Fund will help those hospitals who help themselves, and since common action is more impressive than individual effort, at least in London, the advisability of following a combined policy is fairly clear. If it gains a large following and shows a valuable but yet inadequate result, then it will be easier than it is now to convince the Government that the voluntary principle has done all that can be expected of it. Accustomed as we are to "revised" decisions by Ministers, there may still be some chance of another grant next year from the Government if it has enough evidence meantime to show that the *eventual* yield of the new schemes will suffice. To that argument the Exchequer might yield, because it would then know that the limit of calls upon it is assured. As things are, it seems convinced that the voluntary system has another card to play. Those who believe in the system will stake everything on this last card, because if played with reasonable success not only will it secure the future income needed, but perhaps gain another grant from the State to tide over the final emergency of 1922. On every ground, then, the scheme proposed by the Cave Committee and supported by the King's Fund must be put in action without a moment's hesitation or delay.

